# Supplementary material for: Discovery and application of insertion-deletion (INDEL) polymorphisms for QTL mapping of early life-history traits in Atlantic salmon
Source: BMC Genomics. 2010 Mar 8;11:156. doi: 10.1186/1471-2164-11-156 (PMC2838853; doi:10.1186/1471-2164-11-156)
Supplement: Additional file 2 — Information on developed 76 locus single-run INDEL panel in Atlantic salmon. Information on fluorescence labeling, primer concentrations, PCR pooling and links to alignments, INDEL motifs and GENESCAN (Burge and Karlin 1997) predictions of genes/exons are available in html format. [file 1471-2164-11-156-S2.ZIP › Additionalfile2/snpsummary22471.html]

```
Cluster 12870 Contig 1

prev  Summary    Contig List  next
```

Size of Consensus sequence = 724

Number of sequences = 4

Minimum redundancy = 1

Key

A gi|117870130|gb|EG942826.1|EG942826 EST\_ssal\_evf\_26113 ssalevf mixed\_tissue Salmo salar cDNA Salmo salar cDNA clone ssal\_evf\_534\_220\_rev 5', mRNA sequence  
B gi|117851767|gb|EG924463.1|EG924463 EST\_ssal\_evf\_27174 ssalevf mixed\_tissue Salmo salar cDNA Salmo salar cDNA clone ssal\_evf\_536\_008\_fwd 3', mRNA sequence  
C gi|117870131|gb|EG942827.1|EG942827 EST\_ssal\_evf\_26114 ssalevf mixed\_tissue Salmo salar cDNA Salmo salar cDNA clone ssal\_evf\_534\_220\_fwd 3', mRNA sequence  
D gi|117851766|gb|EG924462.1|EG924462 EST\_ssal\_evf\_27173 ssalevf mixed\_tissue Salmo salar cDNA Salmo salar cDNA clone ssal\_evf\_536\_008\_rev 5', mRNA sequence

9 SNPs detected

A B C D  cosegregation weighted

381 - A - A   7/9 77.78
413 - A - A   7/9 77.78
612 - T - T   7/9 77.78
613 - A - A   7/9 77.78
614 - T - T   7/9 77.78
615 - A - A   7/9 77.78
616 - G - G   7/9 77.78
672 G A A A   2/9 22.22
708 . G . C   2/9 11.11
